# miR-631 Inhibits Intrahepatic Metastasis of Hepatocellular Carcinoma by Targeting PTPRE

**DOI:** 10.3389/fonc.2020.565266

**Published:** 2020-12-04

**Authors:** Bingqing Chen, Zhibin Liao, Yongqiang Qi, Hongwei Zhang, Chen Su, Huifang Liang, Bixiang Zhang, Xiaoping Chen

**Affiliations:** ^1^ Hepatic Surgery Center, Tongji Hospital, Tongji Medical College, Huazhong University of Science and Technology, Wuhan, China; ^2^ Hubei Key Laboratory of Hepato-Pancreato-Biliary Diseases, Science and Technology Department of Hubei Province, Wuhan, China; ^3^ Key Laboratory of Organ Transplantation, Ministry of Education, NHC Key Laboratory of Organ Transplantation, Key Laboratory of Organ Transplantation, Chinese Academy of Medical Sciences, Wuhan, China

**Keywords:** miR-631, receptor-type protein tyrosine phosphatase epsilon, tumor suppressor, hepatocellular carcinoma, intrahepatic metastasis

## Abstract

MicroRNAs (miRNAs) have been reported to play critical roles in the pathological development of hepatocellular carcinoma (HCC), one of the most common cancers in the world. Our study aims to explore the expression, function and mechanism of miR-631 in HCC. Our findings are that expression of miR-631 is significantly down-regulated in HCC tissue compared with that in adjacent non-cancerous tissue, and low expression of miR-631 in HCC tissue is associated with cirrhosis, multiple tumors, incomplete tumor encapsulation, poor tumor differentiation, and high TNM stage. Our test results showed that miR-631 could inhibit migration, invasion, epithelial–mesenchymal transition (EMT) and intrahepatic metastasis of HCC. Receptor-type protein tyrosine phosphatase epsilon (PTPRE) as a downstream target of miR-631 could promote migration, invasion and EMT of HCC cells. Besides, the expression of PTPRE had a negative correlation with the expression of miR-631 both *in vivo* and *in vitro*, and increasing expression of PTPRE could reverse inhibitory effects of miR-631 in HCC cells. In sum, our study first demonstrated that miR-631 targeted PTPRE to inhibit intrahepatic metastasis in HCC. We gain insights from these findings into the mechanism of miRNAs regulation in HCC metastasis and further introduce a novel therapeutic target for HCC treatment.

## Introduction

Hepatocellular carcinoma (HCC) is one of the most common cancers in the world, and it is the fourth driver of cancer-related mortality (1). Most of the HCC patients are diagnosed at advanced stages because they have few symptoms early, many of whom have suffered intrahepatic metastasis and lung metastasis, meaning some of them hardly have the chance to accept radical operation. For advanced HCC cases, moreover, the recurrence rate is nearly 80% with the patients, whose 5-year survival rate is only 25–39% (2). In conclusion, metastasis, recurrence and lack of more effective therapy constitute obstacles against HCC treatment, thus it is critical to find some new therapeutic targets from exploring molecular mechanisms of HCC metastasis.

microRNAs (miRNAs) are short (20–24 nt) and conservative non-coding RNAs, which can play big roles in regulating the post-transcriptional level of gene expression by binding the 3′-untranslated regions (3′-UTRs) of mRNAs and consequently interfering with both stability and translation of mRNAs (3). Over the decades, many studies have proved that miRNAs were dysregulated in HCC and could contribute to tumorigenesis and metastasis of HCC. For instance, some miRNAs, such as miR-221 and miR-25, were up-regulated in HCC tissue and could induce tumorigenesis of HCC (4, 5). On the contrary, let-7 and miR-214 were reported functioning as suppressive factors in HCC (6, 7). We searched miRNA profiles of HCC metastasis in dbDEMC 2.0, and we found GSE26323 of Gene Expression Omnibus (GEO) had compared the expression of miRNAs between primary HCC tissue and metastasis tissue (8, 9). Then we selected miR-631, which was down-regulated in HCC metastasis tissue compared with that in primary HCC tissue (LogFC = −2.67, P = 0.005), as the research target in our following efforts. Some studies have shown that miR-631 could inhibit migration and invasion of prostate cancer cells, resensitize bortezomib-resistant multiple myeloma cell lines, and increase bovine embryo development (10–12). However, miR-631 has never been reported in literature specific to HCC.

In our study, we selected miR-631 to be the research target through bioinformatics tools and aimed to explore the function and mechanism of miR-631 in HCC treatment. We detected the expression of miR-631 in HCC tissue and non-cancerous tissue (ANT) and analyzed their clinicopathologic characteristics and prognosis, respectively. Then we explored the function of miR-631 in HCC from *in vitro* and *in vivo* experiments and searched the mechanism of it. Our study aims to explore the expression, function and mechanism of miR-631 in HCC treatment and unveil the potential value of miR-631 as a new therapeutic target in HCC treatment.

## Materials and Methodology

### Patients and Specimens

Some 64 liver tissue samples were taken from HCC patients who underwent hepatectomy at the Hepatic Surgery Center, Tongji Hospital of Huazhong University of Science and Technology (Wuhan, China) during June 2014 and January 2015. These samples were stored at −80°C. The *in vivo* sampling was approved by the Ethics Committee of Tongji Hospital, and the study was arranged following our vow of the Declaration of Helsinki Principles. We kept following patients up until December 31, 2019.

### Cell Lines, Media and Culturing Environment

Huh7, MHCC97-L and HLF cells were received from the China Center for Type Culture Collection (CCTCC, Wuhan, China). BEL-7402 and HEK293T cells were received from the Hepatic Surgery Center of Tongji Hospital and identified by using the STR genotyping test (Genechem Co., Ltd, Shanghai, China).

These cells were cultured using Dulbecco’s modified Eagle’s medium (Invitrogen Corporation, Carlsbad, CA, USA) with 10% fetal bovine serum (Life Technologies Inc., Gibco/Brl Division, Grand Island, NY, USA) in a humid culture room (5% CO_2_/37°C).

### Plasmid Construction

Using psiCHECK-2, we constructed vectors which participated in a luciferase reporter assay. PTPRE WT1, PTPRE MUT1, PTPRE WT2 and PTPRE MUT2 were synthesized by TsingKe (Wuhan, China). pLenti-CMV-puro was used to establish stably overexpressed miR-631. The coding sequence of the PTPRE gene was amplified by PCR and then subcloned into pCDNA3.1 to establish pCDNA3.1-PTPRE, while pCDNA3.1 was used as control. These sequences are summarized in **Supplementary Table 1**.

### Cell Transfection and Transduction

miR-631 mimic, negative control mimic, miR-631 inhibitor, negative control inhibitor and PTPRE siRNA were brought from RiboBio (Guangzhou, China). All oligonucleotides and plasmids were transfected into cells using Lipofectamine 3000 (Invitrogen). To obtain stable cell lines that could overexpress miR-631, BEL-7402 cells were transduced with lentivirus for 24h and then selected from culture media containing 5 μg/ml puromycin (Cayman Chemical Company, Ann Arbor, MI, USA) for 14 days.

### Quantitative Real-Time Polymerase Chain Reaction (qRT-PCR)

Tissues stored in liquid nitrogen was ground into powders and added with TRIzol solution (Thermo Fisher Scientific, MA, USA), or added TRIzol solution into cells rinsed with 4 °C PBS, then pipetted the mixture to homogenize it. We used miRcute miRNA Isolation Kit (Tiangen, Beijing, China) to isolate total miRNA. For total RNA, after adding TRIzol solution, we incubated the mixture for 10 min and added chloroform into it to further mix, and then incubated the mixture for 5 min. We centrifuged the mixture for 15 min at 12,000*g*/4°C. We transferred the aqueous phase to a blank test tube and mixed it with isopropanol. After incubating for 10 min, we centrifuged the mixture for 10 min at 12,000*g*/4°C and discarded the supernatant. We used 75% ethanol to wash the sediment before centrifuging for 5 min at 12,000*g*/4°C. We discarded the supernatant and air-dried the sediment for 5 min. After adding RNase-free water to resuspend the pellet, we derived total RNA.

For miRNA, miRcute Plus miRNA First-Strand cDNA Synthesis kits (Tiangen, Beijing, China) were used for reverse transcription. The second step was completed using miRcute Plus miRNA qPCR Detection Kits (Tiangen, Beijing, China). For mRNA, reverse-transcription system kits (Toyobo, Osaka, Japan) were used to complete reverse transcription. qPCR analysis could be made with standard SYBR Green PCR kits (Toyobo, Osaka, Japan). Small RNA RNU6B (U6) (RiboBio, Guangzhou, China) was used as a control for the expression of miRNA and the GAPDH (RiboBio, Guangzhou, China) was used for the mRNA. The miDETECT a trackTM miR-631 forward primer was brought from RiboBio (Guangzhou, China). PTPRE mRNA primer sequences are summarized in **Supplementary Table 1**.

### Wound Healing Assays

Wound healing assays were conducted in 6-well plates with 1 × 10^6^ cells per plate. After the cells grown to 95% confluence, we used a pipette tip to scratch the plate, and observed the wound at 0 and 48 h, respectively. Transwell assays including migration and invasion tests were conducted in 24-well plates.

### Transwell Assays

For migration assays, we added DMEM to incubate the upper chamber of a Transwell for 0.5 h before plated cells. After that, we re-suspended cells with DMEM to 1 × 10^5^ cells/ml, and added 200 μl in the upper chamber, while adding DMEM with 5% fetal bovine serum in the nether layer. After cell penetration for 24 h, we scrubbed the cells on the upper chamber membrane, then fixed the chamber in 4% paraformaldehyde for 10min and dyed the chamber in 0.1% crystal violet for 10 min. The invasion assays were conducted by pre-coating with 20% Matrigel (BD Biosciences, NJ, USA) diluted with DMEM in the upper chamber of a Transwell 2h earlier before plated cells and adjusted the concentration of resuspension to 2 × 10^5^ cells/ml. Other steps were the same as the migration assays. Cell counts are the average of cells per visual field.

### Western Blot Assay

Tissues stored in liquid nitrogen were ground into powder or discarded the growth media and washed the cells using 4 °C PBS. After removing PBS, we added 4 °C lysis buffer containing RIPA buffer, aprotinin and leupeptin to lyse cells for 30 min in ice. We scraped the cell culture dish and transferred the mixture into a test tube, then centrifuged it for 15 min at 12,000*g*/4°C. The supernatant was total protein.

Briefly, BCA protein assay kits (Bio-Rad, Hercules, CA, USA) were used to measure protein concentrations. Proteins of equal total amounts were separated electrophoretically in 10% SDS-PAGE. Then the proteins were transferred to PVDF membranes (Millipore, Billerica, MA, USA) from gels. The membranes were soaked into Tris-buffered saline containing 0.1% Tween-20 (TBST) with 5% non-fat milk for blocking 2 h. After that, we incubated the PVDF membranes at 4 °C for more than 8 h with primary antibodies of PTPRE (Proteintech Group inc. CHI, USA) and GAPDH (Cell Signaling Technology, Danvers, MA, USA). Secondary antibodies were used to incubate the membranes the next day for 2 h and then we used an enhanced chemiluminescence system (EMD Millipore, Darmstadt, Germany) to get the results.

### Luciferase Reporter Assay

psiCHECK-2-vectors were constructed. 1 × 10^5^ of HEK293T cells per well were added into 24-well plates and cultured for 24 h before being transfected. Then cells were co-transfected with 0.4 μg psiCHECK-2 vector named PTPRE WT1, PTPRE MUT1, PTPRE WT2, or PTPRE MUT2, and 50 nM miR-631 or control mimic using Lipofectamine 3000 (Invitrogen). After being transfected for 48 h, Firefly and Renilla luciferase activities were measured with DualGlo Luciferase Assay System (Promega, USA).

### HCC Orthotopic Implantation

Four-week-old male nude mice purchased from HFK BioScience (Beijing, China) were housed under specific pathogen-free (SPF) conditions, and then bred as per Institutional Laboratory Guidelines for Animal Care. BEL-7402-control and BEL-7402-overexpress miR-631 cells (1 × 10^6^) were suspended with 100 μl DMEM and injected subcutaneously into the flanks of nude mice. After 4 weeks, we anatomized the mice and removed the tumors, cut the tumors into small tissues of approximately 1 mm^3^, then transplanted them into the livers of nude mice (six mice per group) (13). Some 7 weeks later, liver tissues of the nude mice were dissected and fixed. All animal experiments complied with the ARRIVE (Animal Research: Reporting of *In Vivo* Experiments) guidelines.

### Statistical Analyses

GraphPad Prism 5.0 (GraphPad Software, San Diego, CA, USA) and SPSS 22.0 (SPSS Inc., Chicago, IL, USA) software were used for statistical analyses. Quantitative data were analyzed by two-tailed paired or unpaired Student’s t-test. Categorical data were analyzed by Chi-square or Correction Chi-square test. The log-rank test was conducted for survival analysis, and univariate and multivariate Cox hazard analyses were conducted to evaluate the risk factors of mortality. P <0.05 was assumed as a statistically significant difference.

## Results

### miR-631 Is Down-Regulated in HCC Tissues

To explore the valuable miRNA in HCC, we searched HCC metastasis miRNAs profiles in the database dbDEMC 2.0, and found the data in GEO serial number was GSE26323, indicating that miR-631 was down-regulated in HCC metastasis tissue compared with primary HCC tissue (LogFC = −2.67, P = 0.005). This finding meant miR-631 might contribute to the metastasis of HCC. Some studies have shown that miR-631 could inhibit migration and invasion of prostate cancer cells, indicating miR-631 might be a suppressor in other cancers, but it had not been reported in literature specific to HCC.

**Figure 1:**
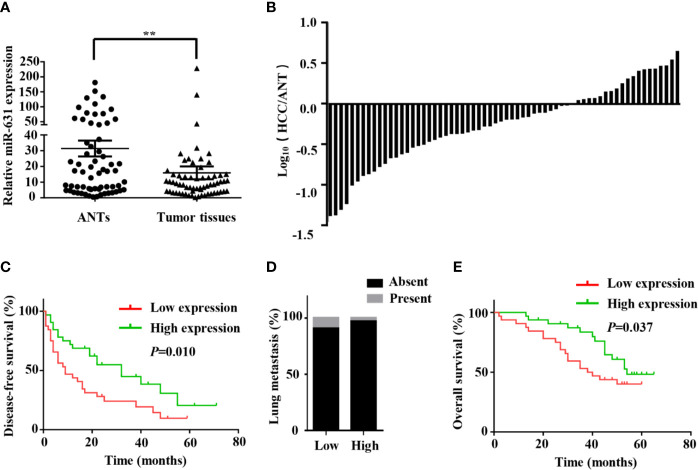
miR-631 is downregulated in tumor tissues of HCC. **(A)** miR-631 expressions in 64 paired HCC tissues and ANTs, measured by qRT-PCR and unit of U6. The data were analyzed by the delta Ct method on a logarithmic scale and compared by paired Student’s t-test. **(B)** The bars represent relative miR-631 expression with the ratio of its level in HCC tissue versus ANT in a logarithmic scale of 64 paired HCC patients. **(C)** Kaplan–Meier survival curves showed that the expression of miR-631 was associated with the disease-free survival of HCC. **(D)** There is no significant difference between the expression of miR-631 and lung metastasis. **(E)** The expression of miR-631 was associated with the overall survival of HCC analyzed by Kaplan–Meier survival curves. **P < 0.01.

In order to make clear miR-631 expression in HCC tissue, we detected its expression in 64 HCC patients, including HCC tissue and adjacent non-cancerous tissue (ANT) by means of qRT-PCR. First, the expression of miR-631 was normalized with that of U6, and we calculated relative miR-631 expression in HCC tissue and ANT in a logarithmic scale of 64 HCC patients. The results indicated that miR-631 expression in HCC tissue was significantly different from that in ANT (**Figure 1A**). Then we normalized miR-631 expression of HCC tissue with that of ANT and derived the ratio in a logarithmic scale of 64 HCC patients. The results showed that miR-631 expression of HCC tissue was lower than that of ANT in 44 HCC patients, and 20 patients had high miR-631 expression in HCC tissue, meaning that miR-631 expression in HCC tissue was significantly lower than that in ANT (**Figure 1B**).

### miR-631 Expression Is Associated With Intrahepatic Metastasis and Prognosis of HCC

The 64 HCC patients were separated into two groups by the median of miR-631 expression in HCC tissues. The low expression group included 32 patients who had low miR-631 levels in HCC tissues. And other 32 patients were separated into high expression group. The clinicopathological analysis showed that patients of both groups had no significant difference in gender, age, alpha-fetoprotein (AFP) expression, Child–Pugh class, and tumor size, but low expression of miR-631 in the tumor was significantly associated with cirrhosis, multiple tumors, incomplete tumor encapsulation, poor tumor differentiation and high TNM stage (**Table 1**).

**Table 1:** Correlations between miR-631 expression and clinicopathologic characteristics in 64 HCC patients.

Variables		miR-631^low^	miR-631^high^	χ^2^	P-value
Gender	Male	27	30	0.642	0.423
	Female	5	2		
Age (years)	≤50	14	20	2.259	0.133
	>50	18	12		
AFP (g/L)	≤20	9	10	0.075	0.784
	>20	23	22		
Cirrhosis	Absent	9	17	4.146	0.042*
	Present	23	15		
Child-Pugh Class	A	24	27	0.869	0.351
	B	8	5		
Tumor number	1	20	27	3.925	0.048*
	≥2	12	5		
Tumor size (cm)	≤5	12	14	0.259	0.611
	>5	20	18		
Tumor encapsulation	Complete	13	22	5.107	0.024*
	Incomplete	19	10		
Tumor differentiation	I–II	18	26	4.655	0.031*
	III–IV	14	6		
TNM stage	I	13	23	6.349	0.021*
	II-IV	19	9		

^*^In bold: The value is statistically significant.

Since multiple tumors, incomplete tumor encapsulation, poor tumor differentiation and high TNM stage were associated with HCC metastasis while tumor size was associated with HCC growth, we speculated that miR-631 is strongly associated with HCC metastasis instead of HCC growth. After analyzing the follow-up data, we found that in the low expression group, the 1-year disease-free survival (DFS) was 43.75%, the 3-year DFS was 24.11% and the 5-year DFS was 9.64%, but in the high expression group, the 1-year DFS was 68.75%, the 3-year DFS was 44.91% and the 5-year DFS was 20.53%, meaning that low expression group had lower DFS than that of high expression group (**Figure 1C**). Moreover, miR-631 expression was not statistically associated with lung metastasis (**Figure 1D**), meaning it might have an important function in intrahepatic metastasis.

Kaplan–Meier log-rank analysis was conducted to explore the correlation between expression of miR-631 and prognosis of HCC patients. The results showed that in the low expression group, the 1-year overall survival (OS) was 90.63%, the 3-year OS was 53.13% and the 5-year OS was 40.1%, but in the high expression group, the 1-year OS was 96.88%, the 3-year OS was 83.53%, and the 5-year OS was 48.26%, meaning HCC patients with the low level of miR-631 expression had lower OS than that of patients with high miR-631 expression (**Figure 1E**).

Since cirrhosis, tumor number, tumor encapsulation, tumor differentiation, and TNM stage were also correlated with HCC prognosis. We stratified these clinicopathologic characteristics to explore whether miR-631 was a prognostic factor of HCC. The results in **Table 2** showed that the expression of miR-631 in patients with cirrhosis, multiple tumor number, and incomplete tumor encapsulation displayed a significant correlation with HCC prognosis. Then we gathered all individual prognostic factors for multivariate analysis (**Table 3**). We found that after considering effects of these prognostic factors, miR-631 was still an independent prognostic factor for OS.

**Table 2:** Univariate stratified cox analysis of prognostic factors.

Variables	Death number (%)		P-value	HR	95.0% CI for HR	
	miR-631^low^	miR-631^high^			Lower	Upper
All cases	21 (66%)	14 (44%)	0.044*	2.012	1.020	3.968
Cirrhosis						
Absent	1 (11%)	7 (41%)	0.106	0.177	0.022	1.447
Present	20 (87%)	7 (47%)	0.001*	5.013	1.933	13.004
Tumor number (>1)						
1	9 (45%)	11 (41%)	0.924	1.044	0.432	2.523
≥2	12 (100%)	3 (60%)	0.019*	6.587	1.372	31.625
Tumor encapsulation						
Complete	6 (46%)	10 (45%)	0.982	0.988	0.358	2.724
Incomplete	15 (79%)	4 (40%)	0.018*	3.841	1.255	11.759
Tumor differentiation						
I–II	9 (50%)	10 (38%)	0.418	1.452	0.589	3.577
III–IV	12 (86%)	4 (67%)	0.199	2.130	0.672	6.748
TNM stage						
I	4 (31%)	9 (39%)	0.520	0.679	0.209	2.210
II-IV	17 (89%)	5 (56%)	0.050	2.795	1.000	7.811

^*^In bold: The value is statistically significant.

**Table 3:** Multivariate analysis of individual prognostic factors.

Variables	P-value	HR	95.0% CI for HR	
			Lower	Upper
miR-631	0.025*	2.401	1.116	5.164
Cirrhosis	0.034*	2.741	1.082	6.947
Tumor number	0.024*	3.220	1.167	8.884
Tumor size (cm)	0.715	1.178	0.490	2.829
Tumor differentiation	0.575	0.764	0.298	1.958
TNM stage	0.135	2.344	0.766	7.170

^*^In bold: The value is statistically significant.

### miR-631 Inhibits Migration and Invasion of HCC Cells

**Figure 2:**
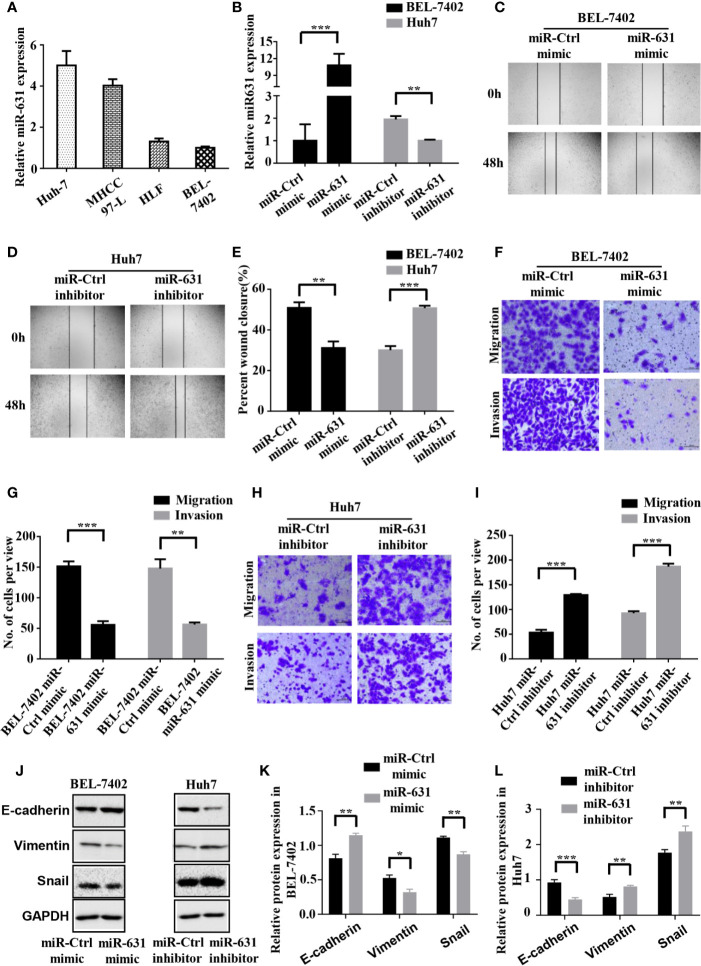
miR-631 inhibits migration and invasion of HCC cells. **(A)** Expression of miR-631 in *in vivo* HCC cell lines. **(B)** Relative expression of miR-631 detected by qRT-PCR in BEL-7402 cells transfected by miR-Control or miR-631 mimic and Huh7 cells transfected by miR-Control or miR-631 inhibitor. The concentration of mimic and inhibitor is 50 nM. **(C–E)** Representative images of wound healing assays and percentages of wound closure were calculated. **(F, G)** Transwell migration and invasion assays of BEL-7402. **(H, I)** Transwell migration and invasion assays of Huh7. **(J–L)** Western blot assay of protein in EMT. Results were represented as mean ± S.E.M. (n = 3) *P < 0.05; **P < 0.01; ***P < 0.001.

We detected the expression of miR-631 in human HCC cell lines including Huh7, MHCC97-L, HLF and BEL-7402 cells. The results showed that Huh7 and MHCC97-L cells had high expression of miR-631 while HLF and BEL-7402 cells had a low expression (**Figure 2A**), which might suggest that the level of miR-631 was potentially related to metastasis since it had high expression in cells of low motility and low expression in cells of high motility. We chose Huh7 and BEL-7402 cells to explore the biological function of miR-631 in HCC cells for further study.

To make the role of miR-631 in HCC migration clear, we carried out cell wound healing assays and Transwell assays. BEL-7402 cells were transfected with miR-Control mimic and miR-631 mimic, while Huh7 cells were transfected with miR-Control inhibitor and miR-631 inhibitor (**Figure 2B**). The images of wound healing assays were shown (**Figures 2C, D**) and the percent of wound closure was calculated, indicating that upregulating miR-631 could decrease the speed of wound closure of BEL-7402 cells, while Huh7 cells with decreased miR-631 expression had a faster wound closure speed than that of control cells (**Figure 2E**).

Then we carried out the Transwell migration assay and invasion assay. The results revealed that after overexpressing miR-631 by transfecting miR-631 mimic, migration and invasion capacities of BEL-7402 cells decreased (**Figures 2F, G**). In contrast, down-regulation of miR-631 expression in Huh7 cells significantly increased the invasion capacities (**Figures 2H, I**). Besides, after regulating the expression of miR-631, we found changes in the expression of epithelial marker (E-cadherin), mesenchymal marker (Vimentin) and transcriptional factor (Snail) as well. The expression of E-cadherin had a positive correlation with the expression of miR-631, while expressions of Vimentin and Snail had negative correlations with miR-631, meaning that miR-631 could inhibit the process of epithelial–mesenchymal transition (EMT), which is widely considered to be crucial to the invasion-metastasis cascade during cancer progression (**Figures 2J–L**). These results proved that miR-631 could inhibit the action of migration and invasion in HCC cells.

### PTPRE Is a Direct Downstream Target of miR-631

**Figure 3:**
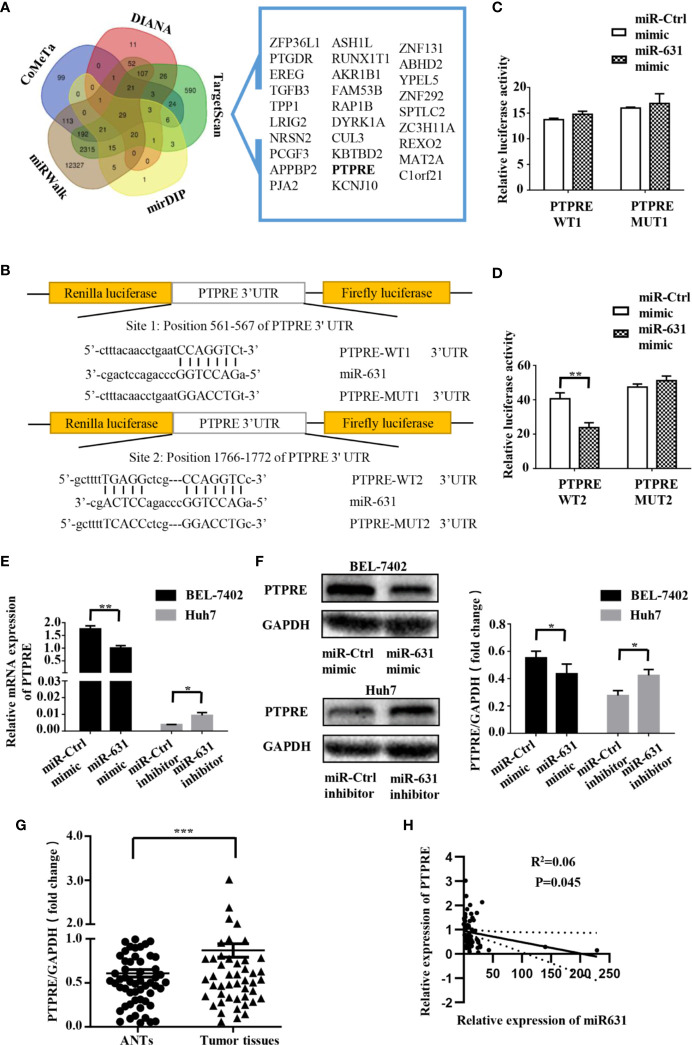
PTPRE is a direct downstream target of miR-631 in HCC cell lines. **(A)** Venn diagram of five databases that predicted downstream target of miR-631. The overlaps are shown. **(B)** The putative binding sites of miR-631 and the corresponding mutant sites in PTPRE 3′-UTR. The diagram showed the positions of sites in psiCHECK-2-vector. **(C, D)** Relative luciferase assay in HEK293 cells where miR-631 mimic was co-transfected with psiCHECK-PTPRE wild-type or psiCHECK-PTPRE mutant vector of sites 1 and 2. **(E, F)** The mRNA and protein expression levels of PTPRE in BEL-7402 and Huh7 cells after being transfected by miR-631 mimic and miR-631 inhibitor, respectively. **(G)** The protein expression of PTPRE in 64 HCC tissues. **(H)** The correlation between the expression of PTPRE and miR-631 in HCC tissues. The concentration of mimic and inhibitor is 50 nM. *P < 0.05; **P < 0.01; ***P < 0.001.

We collected the data of predictive miR-631 targets from five independent databases: DIANA (275 candidate targets) (14), CoMeTa (512 candidate targets) (15), mirDIP (105 candidate targets) (16), miRWalk (15218 candidate targets) (17) and TargetScan (3388 candidate targets) (18), and drew a Venn diagram from them (**Figure 3A**). As shown in the Venn diagram, 29 candidate targets overlapped in the five databases. By analyzing the characteristics and functions of 29 genes, we chose PTPRE for the later study. PTPRE is an isoform of a subfamily of the protein tyrosine phosphatases (PTPs), which plays a role in controlling the reversible phosphorylation of tyrosine residues (19–21). In addition, it had been reported that PTPRE could act as an oncogene in some kind of cancers (22–24), indicating it had an opposite function with miR-631.

To make clear whether PTPRE was a direct target of miR-631, we carried out a dual-luciferase reporter assay. The binding sites of miR-631 and PTPRE were predicted on Targetscan (**Figure 3B**). The reporter vectors contained wild-type or mutated binding sequences, we transfected them into HEK293T cells with miR-control or miR-631 mimic respectively. Data showed that there was no significant difference in the PTPRE-WT1 group after being transfected by miR-631 mimic (**Figure 3C**). However, after increasing miR-631 expression, the relative luciferase activity of PTPRE-WT2 group was down-regulated (**Figure 3D**). These results suggested that miR-631 could target mRNA of PTPRE on the binding site 2 directly.

By increasing miR-631 expression in BEL-7402, the level of mRNA of PTPRE was decreased. And the expression of PTPRE mRNA was upregulated after downregulated miR-631 in Huh7 cells (**Figure 3E**). The change of PTPRE protein level followed the expression of PTPRE mRNA (**Figure 3F**). Besides, we detected PTPRE expression in 64 HCC patients, including HCC tissues and ANTs by Western Blot. The results showed that the PTPRE expression in HCC tissue was significantly higher than that in ANT (**Figure 3G**). By comparing the expression of PTPRE with miR-631 in HCC tissues, we found a negative correlation between them (**Figure 3H**).

These findings revealed that miR-631 expression had a negative correlation with mRNA and protein of PTPRE, suggesting that miR-631 did have a certain impact on the PTPRE translation process.

### PTPRE Promotes Migration and Invasion of HCC Cells

**Figure 4:**
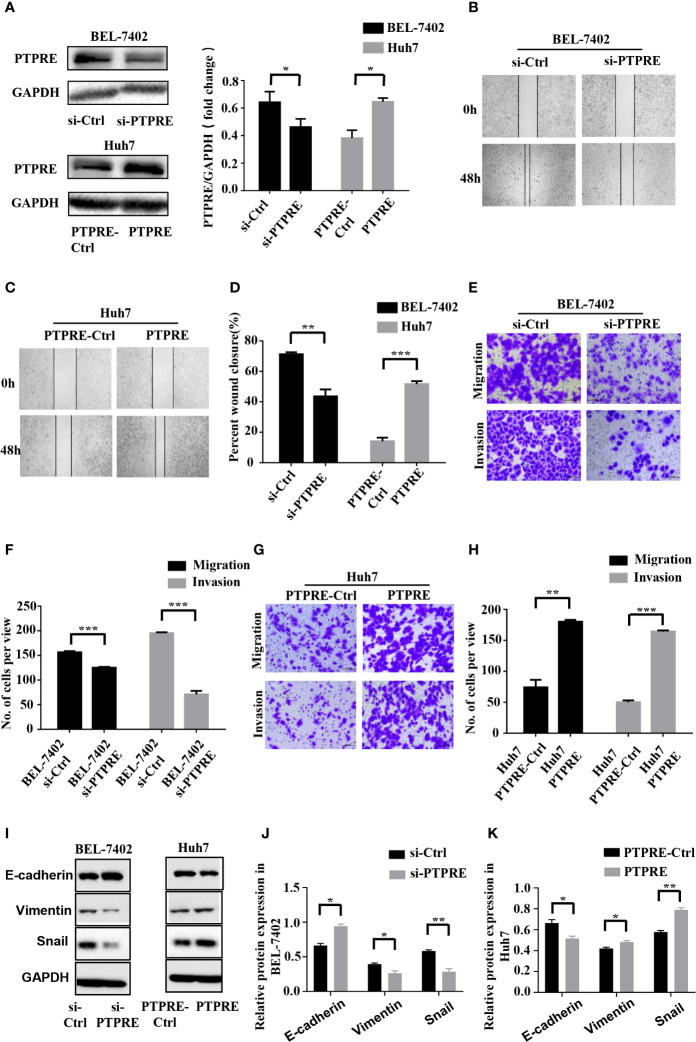
PTPRE induces migration and invasion of HCC cells. **(A)** Western blot assays of PTPRE in BEL-7402 and Huh7 cells after knocking down and overexpressing PTPRE separately. **(B–D)** The representative images of wound healing assay were obtained and the percentages of wound closure were calculated. **(E, F)** Transwell migration and invasion assays of BEL-7402 cells. **(G, H)** Transwell migration and invasion assays of Huh7 cells. **(I–K)** Western blot assay of protein in EMT. *P < 0.05; **P < 0.01; ***P < 0.001.

To investigate the function of PTPRE, we used siRNA to knockdown PTPRE expression in BEL-7402 cells. Huh7 cells, meanwhile, were overexpressed PTPRE from being transfected by pcDNA3.1-PTPRE (**Figure 4A**). Then we proceeded to cell wound healing assays and Transwell assays. The wound healing assay showed cells with a high level of PTPRE had higher percent of wound closure area than cells with a low PTPRE level (**Figures 4B–D**). The Transwell chamber migration and invasion assays showed that the mobility of BEL-7402 cells was decreased after down-regulating PTPRE (**Figures 4E, F**), and high expression of PTPRE could promote migration and invasion of Huh7 (**Figures 4G, H**). Besides, the expression of PTPRE had a negative correlation with the expression of E-cadherin and had positive correlations with expressions of Vimentin and Snail, suggesting PTPRE might induce EMT (**Figures 4I–K**). These findings indicated that PTPRE promoted migration and invasion of HCC cells.

### miR-631 Inhibits Migration and Invasion in HCC Cells Through PTPRE

**Figure 5:**
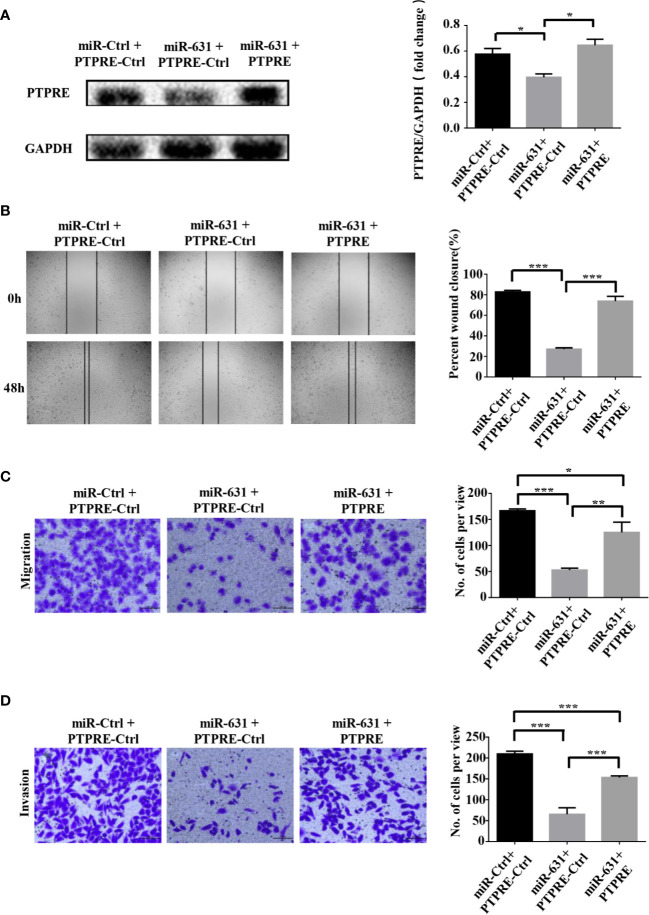
miR-631 inhibits migration and invasion in HCC cells through PTPRE. **(A)** BEL-7402 cells were separated into three groups for carrying out a rescue experiment. **(B)** The representative images of wound healing assay were obtained and the percentages of wound closure were calculated. **(C, D)** Transwell migration and invasion assays of rescue experiment. *P < 0.05; **P < 0.01; ***P < 0.001.

We carried out a rescue experiment to further demonstrate that miR-631 inhibited HCC migration and invasion by targeting PTPRE. We separated BEL-7402 cells into three groups. Cells of the control group were transfected by miR-Control mimic and pcDNA3.1-Control. Cells of the miR-631 overexpression group were transfected by miR-631 mimic and pcDNA3.1-Control. And cells of the high expression of miR-631 and PTPRE group were transfected by pcDNA3.1-PTPRE and miR-631 mimic (**Figure 5A**). The cell wound healing assays showed that the reduced percentage of cells wound closure area was reversed by up-regulating PTPRE (**Figure 5B**). And Transwell migration and invasion assays showed that after increasing expression of PTPRE, the inhibitory effect caused by miR-631 in migration and invasion of BEL-7402 cells was partially reversed (**Figures 5C, D**). These results provided evidence that miR-631 could act as a tumor suppressor by inhibiting PTPRE-enhanced migration and invasion in HCC cells.

### miR-631 Could Inhibit Intrahepatic Metastasis of HCC

**Figure 6:**
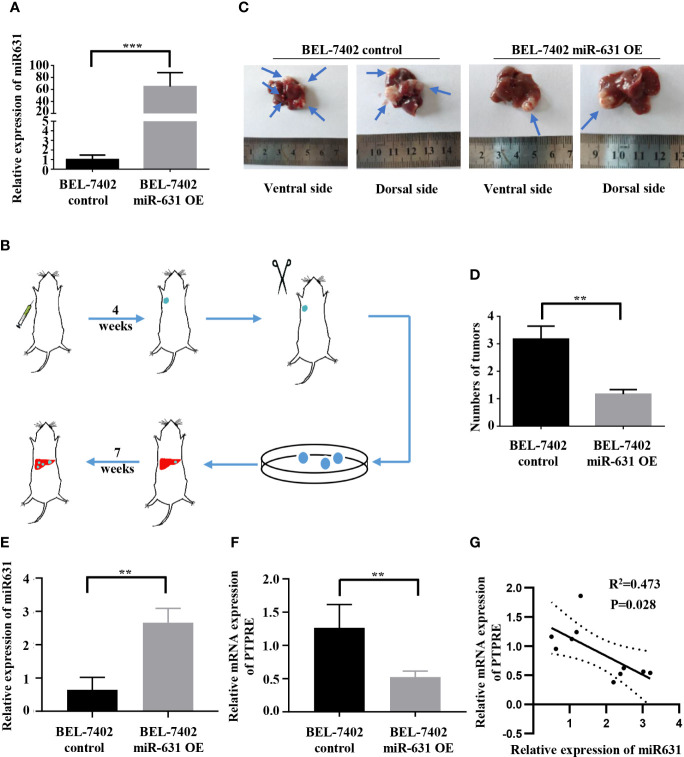
miR-631 inhibits tumor intrahepatic metastasis *in vivo*. **(A)** Relative expression of miR-631 detected by qRT-PCR in BEL-7402 control and BEL-7402 miR-631 OE cells which stably overexpressed miR-631 (n = 3). **(B)** Schematic illustration of *in vivo* metastasis mouse model. **(C)** Images of intrahepatic metastasis tumors of BEL-7402 control and BEL-7402 miR-631 OE. **(D)** The tumor count in livers of BEL-7402 control and BEL-7402 miR-631 OE (n = 6). **(E)** The relative expression of miR-631 in HCC tissues of mice models. **(F)** The relative mRNA expression of PTPRE in HCC tissues of mice models. **(G)** The correlation between miR-631 and mRNA of PTPRE in HCC tissues of mice models. **P < 0.01; ***P < 0.001.

To test *in vivo* function of miR-631 in HCC metastasis, we used lentivirus to construct BEL-7402 cells that could stably overexpress miR-631 (**Figure 6A**) and a mouse model. First, we conducted an *in vivo* tumorigenesis assay. After the tumor diameter was near 1 cm, we cut the tumor tissue into pieces approximate 1 mm^3^ and transplanted them into livers of nude mice. Some 7 weeks later, mice were anatomized (**Figure 6B**), and the liver tissues showed that those with low expression of miR-631 were easier to have intrahepatic metastasis than those of high miR-631 level (**Figures 6C, D**), meaning that miR-631 was able to inhibit intrahepatic metastasis.

We detected the expression of miR-631 and the mRNA level of PTPRE in liver tissues of nude mice (**Figures 6E, F**). The results indicated a negative correlation between the expression of miR-631 and PTPRE mRNA in HCC tissues of mice models (**Figure 6G**).

## Discussion

Since the first miRNA was discovered in 1993 (25), a myriad of miRNAs had been researched and extensive studies revealed that miRNAs could contribute to the progression of a lot of cancers including HCC. In our study, we found that the expression of miR-631 was lower in HCC tissue than that in ANT. The analysis of clinicopathological and prognostic features revealed that patients with low expression of miR-631 were significantly associated with cirrhosis, multiple tumors, incomplete tumor encapsulation, poor tumor differentiation, high TNM stage, short disease-free survival time and short overall survival time, and had no significant difference in gender, age, alpha-fetoprotein (AFP) expression, Child–Pugh class, tumor size, and lung metastasis. From these results, we found that miR-631 was strongly associated with HCC metastasis, especially intrahepatic metastasis. Kaplan–Meier log-rank analysis showed that HCC patients with a low level of miR-631 expression had lower OS than that of patients with high miR-631 expression. Univariate stratified cox hazard analysis was used to evaluate prognostic factors while excluding the impact of some clinicopathologic characteristics (**Table 2**). We found that the expression of miR-631 was correlated with prognosis in HCC patients with cirrhosis, multiple tumor number, and incomplete tumor encapsulation. Multivariate analysis was then used to evaluate the influence of individual prognostic factors, including miR-631, cirrhosis, tumor number, tumor size, tumor differentiation, and TNM stage. The results indicated that the expression of miR-631 was still an independent prognostic factor for HCC OS.

Some studies had shown that miR-631 could inhibit the mobility of migration and invasion of prostate cancer cells by targeting Zeta chain of T cell receptor-associated protein kinase 70, meaning miR-631 might be a biomarker to reveal the capacity of tumor metastasis. However, more clinical supports are still needed.

Our speculations above were also confirmed in terms of expression levels of miR-631 in several HCC cell lines. miR-631 had low expressions in cells with high invasion ability and had high expressions in cells with low invasion ability, meaning the level of miR-631 was potentially associated with HCC metastasis. And the cell wound healing assays and Transwell assays showed that miR-631 played an important role in the motion, migration, and invasion of HCC cell lines. Western blot assay indicated that the expression of miR-631 had a positive correlation with E-cadherin level and had negative correlations with expressions of Vimentin and Snail, suggesting miR-631 might inhibit the process of EMT.

Next, we analyzed the data on predicted miR-631 targets from five independent databases. And we proved that miR-631 could bound with the second predicted binding site of PTPRE mRNA by Dual-luciferase reporter assay. Changing miR-631 expression in HCC cells could make a reverse effect on the expression level of PTPRE mRNA and protein both in HCC cell lines and HCC tissues. Inhibiting translation is the most important function of miRNAs acting in biological processes and it includes two parts: initiation step and post-initiation step. At the initiation step, miRNAs restrained ribosomes from binding to the 5′-cap structure of mRNAs (26–28). And miRNAs could target mRNA in the polysome fraction at the post-initiation stage (29–32). The two interactions might be the reason why miR-631 could regulate the expression of PTPRE mRNA PTPRE proteins. However, more efforts are still needed to explore the in-depth mechanism of the interaction between miR-631 and PTPRE mRNA.

It’s reported that in hepatocytes and liver, PTPRE inactivates insulin receptor signaling (33), which might influence both risk and prognosis in many kinds of cancers (34, 35). And our laboratory had discovered that PTPRE could activate the transforming growth factor-β (TGF-β) β signaling pathway, meaning it could stimulate the EMT and promote migration and invasion of HCC cells (36–39). In our study, PTPRE was proved to have the ability of promoting cell migration and invasion by wound healing assays and Transwell assays. Western blot assay also suggested that PTPRE might induce EMT. And increasing PTPRE in HCC cells could partially reverse the effects caused by miR-631, meaning other target proteins or signal pathways may need to be explored.

The animal study showed that miR-631 could inhibit intrahepatic metastasis of HCC *in vivo*. Same with the results *in vitro*, the expressions of miR-631 and PTPRE in HCC tissues of mice models were negatively related, meaning that our speculations *in vitro* were confirmed *in vivo* by the animal study.

Our study indicated that low expression of miR-631 in HCC was related to the aggressive tumor and proved that miR-631 participated in the process of EMT and could inhibit HCC migration, invasion and intrahepatic metastasis. Besides, PTPRE, which could induce HCC cell migration, invasion and EMT, was demonstrated to be a direct target of miR-631. The expression of PTPRE had a negative correlation with miR-631 level and upregulating PTPRE could partially reverse the effects caused by a high level of miR-631. To our knowledge, our study for the first time showed that miR-631 had a low expression in HCC tissue and explored the miR-631/PTPRE axis in the progression of HCC. But the rescue experiment revealed that more efforts are still needed to explore other downstream targets. Further studies are required to investigate whether miR-631 can serve as a potential prognostic biomarker of HCC and a new therapeutic target in HCC treatment.

## Data Availability Statement

The original contributions presented in the study are included in the article/**Supplementary Material**. Further inquiries can be directed to the corresponding authors.

## Ethics Statement

The studies involving human participants were reviewed and approved by the Ethic Committee of Tongji Hospital, Huazhong University of Science and Technology. The patients/participants provided their written informed consent to participate in this study. The animal study was reviewed and approved by the Ethic Committee of Tongji Hospital, Huazhong University of Science and Technology.

## Author Contributions

BC and ZL carried on the experiments and analysis of this study. BC, ZL and YQ designed the research. HZ and CS provided administrative supports. BC wrote the manuscript. XC, BZ, and HL revised the manuscript. All authors contributed to the article and approved the submitted version.

## Funding

This research was supported by grants from the National Natural Science Foundation of China (No. 81572855 and No. 81202300) and a project of Hubei Natural Science Foundation of China (No. 2015CFB462).

## Conflict of Interest

The authors declare that the research was conducted in the absence of any commercial or financial relationships that could be construed as a potential conflict of interest.
