# Supplementary material for: miR-631 Inhibits Intrahepatic Metastasis of Hepatocellular Carcinoma by Targeting PTPRE
Source: Front Oncol. 2020 Dec 4;10:565266. doi: 10.3389/fonc.2020.565266 (PMC7746836; doi:10.3389/fonc.2020.565266)
Supplement: Supplementary file 1 [file Table_1.docx]

**Supplementary table 1.** Sequences of all oligonucleotides

| Oligonucleotide | Sequences |
| --- | --- |
| PTPRE WT1 | |
| Forward | 5’-TCGAGCTTTACAACCTGAATCCAGGTCTAAAACACAC  TAGAGGC-3’ |
| Reverse | 5’-GGCCGCCTCTAGTGTGTTTTAGACCTGGATTCAGGTTG  TAAAGC-3’ |
| PTPRE MUT1 | |
| Forward | 5’-TCGAGCTTTACAACCTGAATGGACCTGTAAAACACAC  TAGAGGC-3’ |
| Reverse | 5’-GGCCGCCTCTAGTGTGTTTTACAGGTCCATTCAGGTTG  TAAAGC-3’ |
| PTPRE WT2 | |
| Forward | 5’-TCGAGGCTTTTTGAGGCTCGCCAGGTCCCTTTTGTTTT  CACCAGC-3’ |
| Reverse | 5’-GGCCGCTGGTGAAAACAAAAGGGACCTGGCGAGCCT  CCAAAAAGCC-3’ |
| PTPRE MUT2 | |
| Forward | 5’-TCGAGGCTTTTTCACCCTCGGGACCTGCCTTTTGTTTT  CACCAGC-3’ |
| Reverse | 5’-GGCCGCTGGTGAAAACAAAAGGCAGGTCCCGAGGGT  GAAAAAGCC-3’ |
| OE miR-631 | |
| Forward | 5’-CGGTGCTGAGGTCTGGGCCAGGTCTCTCGAGAGACC  TGGCCCAGACCTCAGCTTTTTG-3’ |
| Reverse | 5’-AATTCAAAAAGCTGAGGTCTGGGCCAGGTCTCTCGA  GAGACCTGGCCCAGACCTCAGCA-3’ |
| PTPRE CDS primer | |
| Forward | 5’-GGATCCATGGAGCCCTTGTGTCCACT-3’ |
| Reverse | 5’-GAATTCCATTTGAAATTAGCATAATCAGAA-3’ |
| PTPRE primer | |
| Forward | 5’-CAGCACCAGCGACAAGAAGAT-3’ |
| Reverse | 5’-CCACGGGGATGGGAAAATACTT-3’ |
